# Epidemiological profile of surgical treatment of varicose veins in Brazil from 2010 to 2020

**DOI:** 10.1590/1677-5449.202102021

**Published:** 2022-11-07

**Authors:** Lívia Guerreiro de Barros Bentes, Rafael Silva Lemos, Deivid Ramos dos Santos, José Maciel Caldas dos Reis

**Affiliations:** 1 Universidade do Estado do Pará - UEPA, Laboratório de Cirurgia Experimental - LCE, Belém, PA, Brasil.

**Keywords:** varicose veins, surgical procedures vascular, epidemiological profile

## Abstract

**Background:**

Varicose veins have become more common over recent years and in the most serious cases surgical treatment is necessary to resolve patients’ clinical status. Despite their importance, there are no epidemiological studies that cover the whole of Brazil, showing how surgery to correct varicose veins conducted by the Unified Health System (SUS) is distributed in the country.

**Objectives:**

To describe the ecological profile of surgical treatment to correct varicose veins in Brazil from 2010 to 2020.

**Methods:**

This is a descriptive-analytical study of data obtained from the SUS Hospital Information System. These data were tabulated and categorized by state, region, type of procedure, and year. BioEstat 5.3 was used to conduct chi-square statistical tests with a 95% confidence interval and significance cutoff of p <0.05.

**Results:**

From 2010 to 2020, 755,752 surgical operations to treat varicose veins were conducted; 292,538 were unilateral (38.71%) and 463,214 (61.29%) were bilateral. Of these, 418,791 (55.41%) procedures were performed in the Southeast region, followed by 180,689 (23.91%) in the South region. A total of 40 deaths were registered in connection with these procedures during the period, 26 of which (65%) were associated with bilateral surgery and the majority of which occurred in the Southeast (24 deaths).

**Conclusions:**

It was observed that the majority of procedures are performed in the Southeast and South regions, and that bilateral elective surgery is the most prevalent.

## INTRODUCTION

Varicose veins manifest as dysfunctional turgid and tortuous veins that primarily involve the lower limbs.^1^ Veins have valves in their lumina that prevent blood from refluxing and maintain it pumping in a single direction. However, when the valve system is dysfunctional, venous return becomes ineffective, culminating in accumulation of blood, with considerable impact on functional and esthetic aspects of patients’ lives.^2^

Development of varicose veins is a sign of chronic venous disease (CVD), which is classified according to the involvement and severity of venous insufficiency, in addition to symptoms such as localized burning sensation, pain, tingling, and irritation.^3^ In Brazil, around 62% of women and 37% of men over the age of 30 have the disease,^1^ which is correlated with genetic factors, incompetent valves, pregnancy, hypertension, local hypoxia, and environmental factors, which increase the risk of vascular complications due to hemorrhages and thromboembolism, if untreated.^4^^-^6

In mild cases, conservative treatment can be used, with drugs, lifestyle changes, and compressive elastic bandages and elastic stockings to facilitate venous return.^7^ However, surgical treatments may be needed for more advanced cases. Nowadays, there are several different treatment methods for varicose veins, which may be indicated in isolation or in combination to achieve better esthetic and functional results.

Thermal ablation functions by destroying dysfunctional veins using heat, of which methods using lasers or radio frequency are the most common. In turn, sclerotherapy with polidocanol foam uses a detergent action to damage the endothelium of involved veins. Finally, there is conventional surgery, which encompasses internal or external saphenectomy, saphenofemoral ligation, and phlebectomy for removal of involved vessels.^8^

Surgical procedures are indicated in patients who are classified as C2 or higher on a scale based on clinical, etiologic, anatomic, and pathological features (CEAP). Patients for whom surgical procedures are indicated may have lower limb edema, turgid superficial veins, ochrodermatitis, and healed or active ulcers.^7^^,^^8^ Surgical treatment aims to resolve cosmetic problems, relieve symptoms, remove incompetent veins, and prevent complications and progression of CVD to more advanced classes.^3^^,^^5^

Surgery is based on saphenectomies, saphenofemoral ligation, and phlebectomies, which may be bilateral or unilateral. These procedures consist of marking varicose veins followed by making small incisions in the patient’s limbs that enable ligature of larger veins before exeresis of dysfunctional branches, which can be performed with hooks or illuminated instruments.^9^ The operation takes about 30 minutes, with low risk of complications, and admission to hospital in unnecessary, since most patients are discharged on the same day as the procedure, depending on the case.^10^

Varicose vein surgery is performed by vascular surgeons and must be conducted at a specialist center with specific instruments, equipment, and materials.^9^^,^^10^ In view of this, it can be assumed that in Brazil this service is predominantly provided in the large capitals, primarily in the more developed regions along the South-Southeast axis, or in metropolitan areas. As has been described by Reis et al.,^11^ 80% of vascular surgeons are concentrated in metropolitan areas. The lack of qualified professionals in places far from the capitals makes access more difficult and may result in development of more advanced forms of CVD, leaving the population without care and increasing the likelihood that the condition will exacerbate.

Despite the epidemiological importance of knowledge about the distribution of this disease, few studies have analyzed the profile of varicose vein surgeries, particularly in Brazil,^1^^,^^5^^,^^11^^,^^12^ which is a vast country with unequally distributed specialist medical care. There are no epidemiological time series studies that encompass all procedures used for varicose vein treatment on the public healthcare network and demonstrate the gaps in availability of medical care for this disease. In view of this, the objective of this study was to survey the profile of surgery conducted for varicose vein treatment in Brazil from 2010 to 2020.

## METHODOLOGY

This study is defined as an ecological study, i.e., it is an aggregate study observing an entire population, not just individuals,^13^^,^^14^ and it was conducted with data collected from the Hospital Information System maintained by the Brazilian Unified Health System (SUS - Sistema Único de Saúde), available on the Ministry of Health website TABNET.^13^ The study analyzed all cases of patients who underwent surgical varicose vein treatment from 2010 to 2020. Additionally, mortality data were also accessed using the SUS on-line database.

The data collected were classified as unilateral surgical varicose vein treatments (0406020566), performed on one lower limb only, or bilateral treatments (0406020574), performed on both lower limbs, as classified by the database employed. Data were also tabulated and categorized by region, state, year, mortality, and type of procedure.

To illustrate the incidence of procedures in Brazil, a map of the country’s states was constructed using TabWin v4.15, available on an on-line platform maintained by the SUS IT Department (DATASUS). The coefficient of incidence was distributed by equal frequencies and calculated by dividing the absolute number of procedures in each state or the Federal District by the population resident in that state or district, obtained from population estimates provided by DATASUS, and then multiplied by 10,000.

BioEstat 5.3 was used to perform the chi-square statistical test of adherence, used in analytical and descriptive studies. It was used to analyze the statistical relationships between the regions of Brazil and between the types of procedure performed, adopting a 95% confidence interval and a cutoff for significance of p <0.05.^14^^,^^15^ Since the data analyzed are available from a public domain database, it was unnecessary to submit the study to a Research Ethics Committee.

## RESULTS

From 2010 to 2020, the SUS performed 755,752 varicose vein treatment surgeries, 292,538 of which were unilateral (38.71%) and 463,214 (61.29%) were bilateral. By region, 418,791 (55.41%) procedures were performed in the Southeast, followed by 180,689 (23.91%) in the South, 90,868 (12.02%) in the Northeast, and 24,660 in the North (Table 1).

**Table 1:** Distribution of cases of varicose vein surgeries reported in Brazil by region from 2010 to 2020.

**Varicose vein surgery**	**N**	**%**	**NE**	**%**	**MW**	**%**	**SE**	**%**	**S**	**%**	Total*	**%**
**Unilateral**	5,876	2.01	36,504	12.48	4,099	1.40	129,174	39.96	116,885	39.96	292,538	38.71
**Bilateral**	18,784	4.06	54,364	11.74	36,645	7.91	289,617	13.77	63,804	13.77	463,214	61.29
**Total**	24,660	3.26	90,868	12.02	40,744	5.39	418,791	23.91	180,689	23.91	755,752	100.00

* x² trend test (< 0.0001); N: North; NE: Northeast; MW: Midwest; SE: Southeast; S: South.

Source: Data extracted from the Unified Health System (SUS) Hospital Information System (DATASUS).^13^

**Figure 1:**
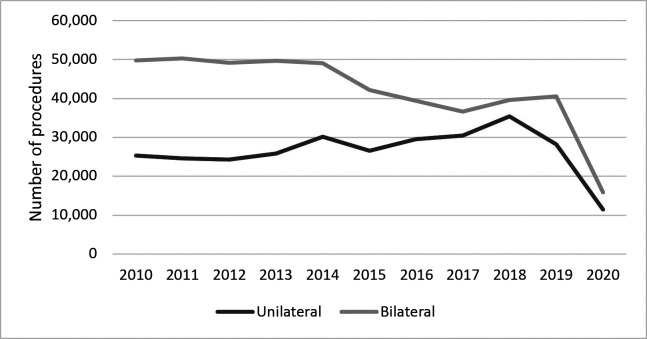
Number of varicose vein surgeries from 2010 to 2020, by year of procedure. Source: Data extracted from the Unified Health System (SUS) Hospital Information System (DATASUS).^13^

By year, the largest number of surgical interventions for varicose veins was in 2014, when 79,212 (12.23%) procedures were conducted, while 2020 was the year with the smallest number of these treatments, with a total of 27,380 (4.22%) cases (Figure 1 and Table 2).

**Table 2:** Distribution of cases of varicose vein surgeries reported in Brazil by region from 2010 to 2020.

**Region**	**2010**	**2011**	**2012**	**2013**	**2014**	**2015**	**2016**	**2017**	**2018**	**2019**	**2020**	Total*	**%**
**North**	1,926	2,453	2,638	5,786	2,972	2,119	1,386	1,318	2,679	911	419	24,607	3.26
**Northeast**	11,321	10,235	9,652	9,970	9,199	7,101	6,964	6,972	7,806	8,046	3,291	90,557	12.01
**Midwest**	4,056	4,856	4,523	4,457	4,270	3,857	3,404	3,309	3,140	3,304	1,487	40,663	5.39
**Southeast**	39,510	40,389	38,751	37,516	43,333	39,282	41,290	39,304	43,016	39,582	15,912	417,885	55.43
**South**	18,282	17,024	17,807	17,769	19,438	16,355	15,879	16,226	18,308	16,885	6,271	180,244	23.91
**Total**	75,095	74,957	73,371	75,498	79,212	68,714	68,923	67,129	74,949	68,728	27,380	753,956	100.00

* x² trend test (< 0.0001).

Source: Data extracted from the Unified Health System (SUS) Hospital Information System (DATASUS).^13^

Total mortality (Table 3) related to the procedure was 40 deaths during the period analyzed, 26 of which were associated with bilateral surgery (65%). By region, the Southeast registered 24 deaths (60%), the Northeast had the second highest mortality, with 9 deaths (22.5%), and the North was the region with fewest deaths during the period, with 1 fatal case (2.5%).

**Table 3:** Distribution of cases of varicose vein surgery mortality reported in Brazil by region from 2010 to 2020.

**Varicose vein surgery**	**N**	**%**	**NE**	**%**	**MW**	**%**	**SE**	**%**	**S**	**%**	Total*	**%**
**Unilateral**	0	0	6	42.85	0	0	7	50	1	7.14	14	35
**Bilateral**	1	3.84	3	11.53	3	11.53	17	65.38	2	7.69	26	65
**Total**	1	2.5	9	22.5	3	7.5	24	60	3	7.5	40	100.00

* x² trend test (< 0.05); N: North; NE: Northeast; MW: Midwest; SE: Southeast; S: South.

Source: Data extracted from the Unified Health System (SUS) Hospital Information System (DATASUS).^13^

**Figure 2:**
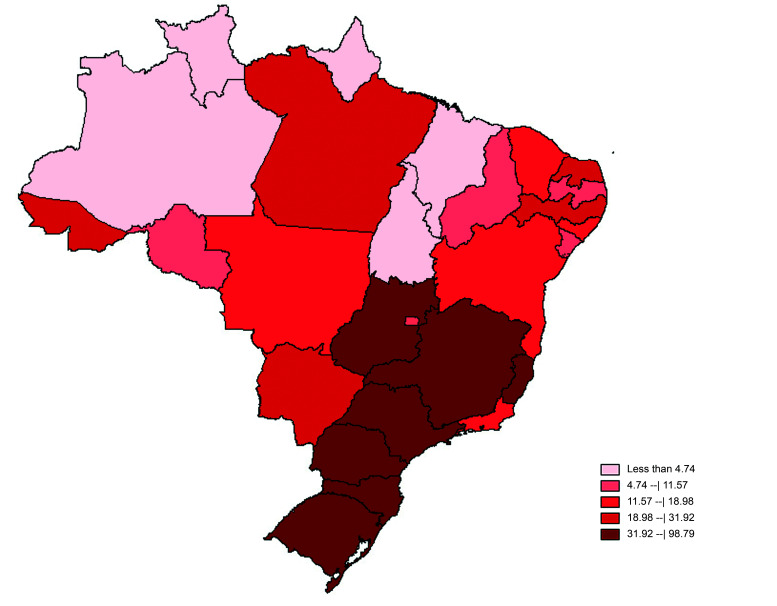
Coefficients of incidence of surgery for varicose veins per 10,000 inhabitants in Brazil from 2010 to 2020. Source: Based on data from the Unified Health System (SUS) Hospital Information System (DATASUS).^13^

A majority of the procedures were elective, accounting for 699,797 (92.59%) surgeries, 266,140 (90.97%) of the unilateral procedures and 433,657 (93.61%) of the bilateral surgeries. The Southeast region had the highest proportion of elective procedures as a proportion of all surgeries (94.61%), while this proportion was lowest in the North, with 88.25% of procedures conducted on an elective basis (Table 4 and Figure 2).

**Table 4:** Distribution of cases of varicose vein surgery reported in Brazil by type of care and region, from 2010 to 2020.

**Varicose vein surgery**	**Type of care**	**N**	**NE**	**MW**	**SE**	**S**	Total*	**%**
**Unilateral**	Elective	4,745	33,060	3,170	121,490	103,675	266,140	90.97
	Urgent	1,131	3,444	929	7,684	13,210	26,398	0.90
**Bilateral**	Elective	17,019	50,240	33,493	274,767	58,138	433,657	93.61
	Urgent	1,765	4,124	3,152	14,850	5,666	29,557	6.38
**Total**	Elective	21,764	83,300	36,663	396,257	161,813	699,797	92.59
	Urgent	2,896	7,568	4,081	22,534	18,876	55,955	7.40

* x² trend test (< 0.0001); N: North; NE: Northeast; MW: Midwest; SE: Southeast; S: South.

Source: Data extracted from the Unified Health System (SUS) Hospital Information System (DATASUS).^13^

## DISCUSSION

Varicose vein surgery is conducted in all regions of Brazil, since it is provided by the SUS at major hospitals and others with the capacity to perform the procedure according to its principals and guidelines.^9^^,^^10^ It is a very important procedure, since chronic venous insufficiency can cause formation of difficult to heal ulcers which may lead to irreparable complications.^12^

The largest numbers of varicose vein surgeries performed during the last 10 years were in the Southeast, the South, and the Northeast regions. The North region contributed little to the numbers, with a little more than 24 thousand procedures, equivalent to 3.26% of cases. In contrast, the Southeast was responsible for 55.41% of the procedures. This constitutes powerful evidence of the access difficulties and the lack of specialist services, primarily in regions far from the country’s metropolitan centers, as in the states in North Brazil. This is possibly because of greater concentrations of specialist physicians in the same regions in which there is greater availability of medium and high complexity services. A target has been set of 2.5 physicians per 1,000 inhabitants, but, in the states of Rio de Janeiro and São Paulo, there are approximately 4.44 and 3.31 physicians per 1,000 inhabitants, respectively. In contrast, few states in the North are close to achieving the minimum level, according to the Federal Medical Council (CFM- Conselho Federal de Medicina).^14^^-^16 The discrepancy in numbers of specialists is even starker, with 3.1 vascular surgeons per 100 thousand inhabitants in São Paulo and 0.5 vascular surgeons per 100 thousand inhabitants in the state of Pará.^11^^,^^16^^,^^17^

Analysis of extent of surgery, unilateral or bilateral, showed that bilateral surgery was more common in all of the regions except the South, where unilateral surgery accounted for 64.68% of the operations performed. This finding corroborates observations reported by Defty et al.,^18^ since patients prefer to undergo a single session of surgery (bilateral) to correct their varicose veins, rather than having to return and undergo the procedure again, subjecting themselves to the risks of anesthesia, admission, and postoperative care.

With regard to the years in which procedures were performed, it was observed that 2014 had the largest number and 2020 the smallest, with a constant decline from 2015 onwards. It is therefore inferred that after 2014 the rate of surgical procedures has been falling because of implementation of alternative treatments for varicose veins on the SUS, such as sclerotherapy with polidocanol foam. For technical, administrative, and structural reasons, it is believed that sclerotherapy has been gaining traction for varicose veins repair, primarily for more advanced cases, because of its cost-benefit ratio, as shown in recent studies.^19^^-^22

Sclerotherapy with polidocanol foam is a simple procedure that is performed with ultrasound guidance and can be used in the superficial and deep veins of the lower limbs, in separate sessions for analysis of progression and administration of the foam.^20^ The procedure is economically more viable for hospitals because it is unnecessary to admit patients, who are discharged after the procedure, and it costs one third of the price of varicose vein surgery.^19^^,^^21^

In a study by Ramussen et al.,^23^ comparison of the outcomes of treatment methods revealed higher rates of recanalization and need for retreatment after sclerotherapy. Despite this, the mean cost of the procedure reported in a study by Epstein et al. was just 249 Euros, compared to a cost of 894 Euros for high saphenous ligature surgery.^21^

With relation to the comparative duration of each procedure, it has been observed that sclerotherapy can be performed in a few minutes, from locating the varicose veins with ultrasound to administration of the foam,^20^ whereas outpatient phlebectomy can take longer, varying from 30 minutes to 5 hours, depending on the numbers of varicose veins.^20^^,^^21^ Sclerotherapy thus enables a vascular clinic to treat many patients on the same day.^24^ Vernemo et al.,^24^ emphasized the postoperative recovery outcomes in a study observing that patients treated with sclerotherapy were free from pain or had minimal pain at 1 week, with earlier return to daily tasks than those treated with other interventions, and achieved the same quality of life and recovery at 1 year follow-up.

As a result of the pandemic caused by the SARS-CoV-2 virus, there was a general reduction in the numbers of non-emergency surgical procedures performed in 2020.^25^ Many types of elective procedures, in addition to vascular surgery procedures, were suspended by health authorities in periods during which the country’s public healthcare network was under greatest stress, because of the high risk of exposure of patients and health professionals and because of the need to transfer resources and transfer hospital infrastructure to treatment of patients infected by the virus.^26^ Furthermore, the patients themselves were worried about contamination by the pathogen, reducing their adherence to procedures.^24^^,^^27^

Another important point to highlight is the mortality associated with the procedure, with 40 deaths during the period from 2010 to 2020, constituting a mortality rate of 5.3 deaths per 100 thousand procedures. Mortality was low in all regions, with differences between numbers primarily linked to the disparities in the numbers of varicose vein surgeries performed in different regions, with higher mortality in the Northeast (9.93/100 thousand) and Mid-West (7.37/100 thousand), linked to distribution of vascular services and surgeons around the country.^11^^,^^12^ Possible trigger factors of death from varicose vein surgery include sepsis, cardiac infection, stroke, deep venous thrombosis, thromboembolism, and pulmonary embolism, showing that although the procedure has low mortality,^28^ it is not risk free and needs specialist care.

A study by Kim et al.^29^ reported similar data to those found in the present study. Mortality was low (0.02%), with no age-linked differences, although elderly people were more likely to suffer complications and adverse events, which were reduced with postoperative care. Another study, conducted in Brazil, observed a rate of 4.52 procedures per 10 thousand inhabitants and a very low mortality rate of 0.0056%.^30^

Additionally, with regard to type of procedure, elective surgery was most prevalent in all regions, since these are procedures that are scheduled and provided by the SUS. This is because even when patients have venous insufficiency and ulcers they are not classified as urgent, so they are defined as qualifying for elective procedures.^31^

The principal limitation of this study is the underreporting that is part of all study designs based on secondary databases, particularly with relation to remote regions far from the large capitals, where there is undoubtedly hidden demand because of the lack of specialist medical care, meaning that cases cannot be reported and care voids occur. Another source of underreporting bias is procedures conducted via private healthcare, which are not registered on the database used in this study. As such, the results obtained may not reflect the situation in this population which, in general, has different socioeconomic and cultural status to the population cared for by the public healthcare network.^1^^,^^11^

Additionally, the possibility of incorrect classifications should also be acknowledged, particularly with relation to varicose vein surgery listed as cause of deaths that could have actually been caused by procedures or actions that are unrelated to surgery for varicose veins. Another limitation present during the study is the absence of data on non-esthetic sclerotherapy on the DATASUS database, limiting the possibilities for cross-referencing of data and correlation with outpatients phlebectomy.

This study aimed to trace the profile of surgery for treatment of varicose veins, recording its characteristics and the regions where it is performed, demonstrating relationships between several different factors and surgery for varicose veins and possible reasons for the reduction in its use over time and also the characteristics of the technique employed.

## CONCLUSIONS

In this study, it was observed that the great majority of varicose vein surgeries are bilateral, elective, and concentrated in the South, and Southeast regions. It is inferred that implementation of more accessible alternative treatments, such as sclerotherapy, may explain the gradual reduction in surgical procedures over the years.

## References

[1] Lins EM, Barros JW, Appolônio F, Lima EC, Barbosa M, Anacleto E (2012). Perfil epidemiológico de pacientes submetidos a tratamento cirúrgico de varizes de membros inferiores. J Vasc Bras.

[2] Jacobs BN, Andraska EA, Obi AT, Wakefield TW (2017). Pathophysiology of varicose veins. J Vasc Surg.

[3] DePopas E, Brown M (2018). Varicose Veins and Lower Extremity Venous Insufficienc. Semin Intervent Radiol.

[4] Raetz J, Wilson M, Collins K (2019). Varicose veins: diagnosis and treatment. Am Fam Physician.

[5] Wu N, Chen Z, Feng I (2020). Severe varicose veins and the risk of mortality: a nationwide population-based cohort study. BMJ Open.

[6] Serra R, Ielapi N, Bevacqua E (2018). Haemorrhage from varicose veins and varicose ulceration: a systematic review. Int Wound J.

[7] Belramman A, Bootun R, Lane TRA, Davies AH (2019). Endovenous management of varicose veins. Angiology.

[8] Rocha FA, Lins EM, de Almeida CC (2020). Quality of life assessment before and after surgery for lower limb varicose veins. J Vasc Bras.

[9] Geersen DF, Shortell CEK (2018). Phlebectomy techniques for varicose veins. Surg Clin North Am.

[10] Barros BCS, Araujo AL, Magalhães CEV, Barros RLS, Fiorelli SKA, Gatts RF (2015). Efficacy of varicose vein surgery with preservation of the great safenous vein. Rev Col Bras Cir.

[11] Reis JMC, Santos DR, Torres IO, De Luccia N (2021). Vascular surgery in the most populous state in Amazon: socio-professional profile and aspirations of the speciality. J Vasc Bras.

[12] Campoy LT, Ramos ACV, Souza LLL (2020). A distribuição espacial e a tendência temporal de recursos humanos para o Sistema Único de Saúde e para a Saúde Suplementar, Brasil, 2005 a 2016. Epidemiol Serv Saude.

[13] DATASUS (2021). DATASUS.

[14] Abraão LSO, José BMPA, Gomes CBS (2020). Perfil epidemiológico dos casos de leishmaniose tegumentar americana no estado do Pará, Brasil, entre 2008 e 2017. Rev Pan-Amaz Saude..

[15] da Silva AFT, Valente FS, de Sousa LD, Cardoso PNM, da Silva MA, dos Santos DR (2021). Estudo epidemiológico sobre meningite bacteriana no Brasil no período entre 2009 a 2018. Rev Med (São Paulo).

[16] Guedes BAP, Vale FLB, Souza RW, Costa MKA, Batista SR (2019). A organização da atenção ambulatorial secundária na SESDF. Ciênc Saúde Colet.

[17] Scheffer M (2020). Demografia Médica no Brasil 2020..

[18] Defty C, Eardley N, Taylor M, Jones DR, Mason PF (2008). A comparison of the complication rates following unilateral and bilateral varicose vein surgery. Eur J Vasc Endovasc Surg.

[19] Pereira AFB, Mesquita A, Gomes C (2014). Abordagens cirúrgicas no tratamento de varizes. Angiol Cir Vasc.

[20] Belramman A, Bootun R, Lane TRA, Davies AH (2019). Foam sclerotherapy versus ambulatory phlebectomy for the treatment of varicose vein tributaries: study protocol for a randomised controlled trial. J Ayub Med Coll Abbottabad.

[21] Epstein D, Onida S, Bootun R, Ortega-Ortega M, Davies AH (2018). Cost-effectiveness of current and emerging treatments of varicoseveins. Value Health.

[22] Kharl RAK, Khan NI, Pervaiz HK (2019). Foam clerotherapy: an emerging, minimally invasive and safe modality of treatment for varicose veins. J Ayub Med Coll Abbottabad.

[23] Rasmussen L, Lawaetz M, Serup J (2013). Randomized clinical trial comparing endovenous laser ablation, radiofrequency ablation, foam sclerotherapy, and surgical stripping for great saphenous varicose veins with 3-year follow-up. J Vasc Surg Venous Lymphat Disord..

[24] Venermo M, Saarinen J, Eskelinen E (2016). Randomized clinical trial comparing surgery, endovenous laser ablation and ultrasound-guided foam sclerotherapy for the treatment of great saphenous varicose veins. Br J Surg.

[25] Diaz A, Sarac BA, Schoenbrunner AR, Janis JE, Pawlik TM (2020). Elective surgery in the time of COVID-19. Am J Surg.

[26] Motta GR, Leal AC, Amaral MVG, Maia PAV, Duarte MEL, Bähr GL (2021). Impacto das estratégias adotadas para enfrentar a pandemia de COVID-19 em um Instituto Brasileiro de referência em cirurgia de alta complexidade em Ortopedia e Traumatologia. Rev Bras Ortop.

[27] Krasinski Z, Krasinska A, Markiewicz S, Zielinski M (2021). Patients with chronic venous insufficiency in the times of COVID-19 and the risk of thrombus formation - suggestions on conservative treatment of such patients based on the principles of pathophysiology. Pol Przegl Chir.

[28] Bootun R, Davies AH (2016). Long-term follow-up for different varicose vein therapies: is surgery still the best?. Phlebology.

[29] Kim TI, Zhang Y, Guzman RJ, Ochoa Chaar CI (2021). Trends of hospital-based surgery for varicose veins in elderly. J Vasc Surg Venous Lymphat Disord.

[30] Silva MJ, Louzada ACS, Silva MFA, Portugal MFC, Teivelis MP, Wolosker N (2022). Epidemiology of 869,220 varicose vein surgeries over 12 years in Brazil: trends, costs and mortality rate. Ann Vasc Surg.

[31] Sant’Ana SM, Bachion MM, Santos QR, Nunes CABN, Malaquias SG, Oliveira BGRB (2012). Úlceras venosas: caracterização clínica e tratamento em usuários atendidos em rede ambulatorial. Rev Bras Enferm.

