# Supplementary material for: Epidemiological profile of surgical treatment of varicose veins in Brazil from 2010 to 2020
Source: J Vasc Bras. 2022 Nov 7;21:e20210202. doi: 10.1590/1677-5449.202102021 (PMC9639579; doi:10.1590/1677-5449.202102021)
Supplement: Translation

# Translation: Perfil epidemiológico do tratamento cirúrgico de varizes no Brasil no período de 2010 a 2020

**DOI:** 10.1590/1677-5449.202102022

**Authors:** Lívia Guerreiro de Barros Bentes, Rafael Silva Lemos, Deivid Ramos dos Santos, José Maciel Caldas dos Reis

**Affiliations:** 1 Universidade do Estado do Pará - UEPA, Laboratório de Cirurgia Experimental - LCE, Belém, PA, Brasil.

**Keywords:** varizes, procedimentos cirúrgicos vasculares, perfil epidemiológico

## Abstract

**Contexto:**

As varizes são um agravo que vêm aumentando nos últimos anos e, nos casos mais graves, necessitam de tratamento cirúrgico para a resolução do quadro clínico do paciente. Apesar da sua importância, não há trabalhos epidemiológicos que englobem todo o território brasileiro de modo a demonstrar como está a distribuição das cirurgias para a correção desse agravo pelo Sistema Único de Saúde.

**Objetivos:**

Descrever o perfil ecológico do tratamento cirúrgico para a correção de varizes no Brasil no período de 2010 a 2020.

**Métodos:**

O estudo caracteriza-se como descritivo-analítico, mediante a coleta de dados proveniente do Sistema de Informações Hospitalares do Sistema Único de Saúde. Os dados, disponibilizados pela própria plataforma, foram tabelados e categorizados de acordo com o estado, a região, o caráter do procedimento e o ano de realização. O programa BioEstat 5.3 foi utilizado para a realização do teste estatístico de qui-quadrado, sendo adotado o intervalo de confiança de 95% e o valor significativo de p <0,05.

**Resultados:**

No período de 2010 a 2020, foram realizadas 755.752 cirurgias para o tratamento de varizes, sendo 292.538 unilaterais (38,71%) e 463.214 (61,29%) bilaterais. A região Sudeste foi responsável por 418.791 (55,41%) procedimentos, seguida pelo Sul, com 180.689 (23,91%). Foram registradas 40 mortes pelo procedimento no período analisado, das quais 26 ocorreram em cirurgias bilaterais (65%) e a maioria no Sudeste (24 óbitos).

**Conclusões:**

Verificou-se que esse procedimento é realizado majoritariamente nas regiões Sudeste e Sul, com maior prevalência da cirurgia bilateral de caráter eletivo.

## INTRODUÇÃO

As varizes se manifestam por veias disfuncionais túrgidas e tortuosas que acometem principalmente os membros inferiores^1^. Esses vasos possuem valvas em seu lúmen, impedindo que o sangue sofra refluxo ao mesmo tempo que mantêm o único sentido do seu bombeamento. Entretanto, quando o sistema de valvas está disfuncional, o retorno venoso se torna ineficiente, culminando no acúmulo de sangue com importante impacto funcional e estético na vida do paciente^2^.

O desenvolvimento de varizes é um sinal de doença venosa crônica (DVC), a qual é classificada de acordo com o acometimento e gravidade da insuficiência venosa, além de sintomas como queimação, dor, formigamento e irritação localizados^3^. Cerca de 62% das mulheres e 37% dos homens acima de 30 anos no Brasil são atingidos por essa doença^1^, que possui correlação com fatores genéticos, valvas incompetentes, gravidez, hipertensão, hipóxia local e fatores ambientais, os quais aumentam o risco de complicações vasculares por hemorragias e tromboembolismo, caso não sejam tratados^4^^-^6.

Em casos leves, pode ser utilizado o tratamento conservador, com fármacos, mudanças de estilo de vida, ataduras elásticas compressivas e meias elásticas para favorecer o retorno venoso^7^. Já nos casos mais avançados, tratamentos cirúrgicos podem ser indicados. Atualmente, há diversas modalidades de tratamento para varizes, podendo ser indicada isoladamente ou em conjunto para favorecer melhores resultados estéticos e funcionais.

A termoablação tem a função de destruir as veias disfuncionais usando calor, sendo o *laser* e a radiofrequência seus principais representantes. Já a escleroterapia com espuma de polidocanol traz a ação detergente para danificar o endotélio das veias envolvidas. Por fim, há a cirurgia convencional, a qual engloba a safenectomia interna ou externa, a crossectomia e a flebectomia para retirada dos vasos acometidos^8^.

O procedimento cirúrgico é indicado em pacientes que apresentam a classe C2 ou superior da classificação baseada em aspectos clínicos, etiológicos, anatômicos e patológicos (CEAP). Os pacientes indicados ao procedimento cirúrgico podem apresentar edema de membros inferiores, turgescência das veias superficiais, dermatite ocre e úlceras cicatrizadas ou ativas^7^^,^^8^. O tratamento cirúrgico visa à resolução cosmética, ao alívio de sintomas, à retirada das veias incompetentes, à prevenção de complicações e à evolução da DVC para as classes avançadas^3^^,^^5^.

A cirurgia se baseia na realização de safenectomias, crossectomias e flebectomias, que podem ser tanto bilaterais quanto unilaterais. Consiste na marcação das varizes com posterior realização de pequenas incisões nos membros do paciente que possibilitem a ligadura das veias maiores para a posterior exérese das suas ramificações disfuncionais, podendo ser realizada com ganchos ou instrumentos iluminados^9^. A operação dura em torno de 30 minutos, com baixo risco de complicações, e a internação não é necessária, sendo que o paciente recebe alta no dia do procedimento, dependendo do caso^10^.

As cirurgias de varizes são realizadas por cirurgiões vasculares e necessitam de um serviço especializado subjacente, com instrumentos, equipamentos e materiais específicos^9^^,^^10^. Por conta disso, presume-se que a oferta desse serviço seja concentrada nas grandes capitais brasileiras, principalmente nas regiões mais desenvolvidas do eixo Sul-Sudeste ou em regiões metropolitanas. Como descrito por Reis et al.^11^, 80% dos cirurgiões vasculares se concentraram nas regiões metropolitanas. A carência de profissionais especializados em áreas longínquas das capitais pode dificultar o acesso e favorecer o desenvolvimento de quadros mais avançados de DVC, deixando a população desassistida e aumentando a chance de agravar o quadro.

Apesar da importância epidemiológica de se conhecer a distribuição dessa doença, há poucos estudos analisando o perfil das cirurgias de varizes, principalmente no Brasil^1^^,^^5^^,^^11^^,^^12^, um país de vasta extensão territorial e com distribuição desigual da assistência médica especializada. Além disso, não há um trabalho epidemiológico com recorte temporal que englobe todos os procedimentos realizados para o tratamento de varizes na rede pública de saúde de modo a demonstrar as lacunas de assistência médica para essa doença. Diante disso, o objetivo deste estudo foi verificar o perfil da cirurgia para tratamento de varizes no Brasil de 2010 a 2020.

## METODOLOGIA

Este estudo caracteriza-se como um estudo ecológico, ou seja, é um estudo agregado que observa populações como um todo, e não apenas o indivíduo^13^^,^^14^, realizado mediante a coleta de dados proveniente do Sistema de Informações Hospitalares do Sistema Único de Saúde (SIH-SUS), disponível no site do Ministério da Saúde TABNET^13^. Foram analisados todos os casos de pacientes submetidos ao tratamento cirúrgico de varizes no período de 2010 a 2020. Além disso, os casos de mortalidade também foram verificados por meio do sistema de dados *on-line* do Sistema Único de Saúde (SUS).

As informações colhidas foram agrupadas em tratamento cirúrgico de varizes unilateral (0406020566), realizado em apenas um membro inferior, e bilateral (0406020574), realizado em ambos os membros, conforme a classificação presente na base de dados utilizada. Ademais, os dados foram tabelados e categorizados de acordo com região, estado, ano, mortalidade e caráter do procedimento.

Para ilustrar a incidência do procedimento no país, foi construído um mapa dos estados brasileiros no programa TabWin v4.15, disponível na plataforma *on-line* do Departamento de Informática do Sistema Único de Saúde. O coeficiente de incidência foi distribuído por frequências iguais e calculado a partir da divisão do número absoluto de procedimentos em cada unidade federativa pela população residente de cada estado, obtido das estimativas populacionais disponibilizadas pelo Departamento de Informática do Sistema Único de Saúde (DATASUS), posteriormente multiplicado por 10.000.

O programa BioEstat 5.3 foi utilizado para a realização do teste estatístico de qui-quadrado de aderência, utilizado em trabalhos analíticos e descritivos. Ele foi empregado para avaliar a relação estatística entre as regiões do Brasil e entre os tipos de procedimento realizados, sendo adotado o intervalo de confiança de 95% e o valor significativo de p <0,05^14^^,^^15^. Tendo em vista que os dados interpretados estão presentes em banco de dados de domínio público, não foi necessário submeter este estudo ao Comitê de Ética em Pesquisa.

## RESULTADOS

No período de 2010 a 2020, pelo SUS, foram realizadas 755.752 cirurgias para o tratamento de varizes, sendo 292.538 unilaterais (38,71%) e 463.214 (61,29%) bilaterais. Conforme as regiões, o Sudeste foi responsável por 418.791 (55,41%) dos procedimentos, seguido pelo Sul, com 180.689 (23,91%), e Nordeste, com 90.868 (12,02%). O Norte contribuiu com 24.660 cirurgias (Tabela 1).

**Tabela 1:** Distribuição dos casos notificados no Brasil de cirurgias de varizes por região entre os anos de 2010 e 2020.

**Cirurgia de varizes**	**N**	**%**	**NO**	**%**	**CO**	**%**	**SE**	**%**	**S**	**%**	Total*	**%**
**Unilateral**	5.876	2,01	36.504	12,48	4.099	1,40	129.174	39,96	116.885	39,96	292.538	38,71
**Bilateral**	18.784	4,06	54.364	11,74	36.645	7,91	289.617	13,77	63.804	13,77	463.214	61,29
**Total**	24.660	3,26	90.868	12,02	40.744	5,39	418.791	23,91	180.689	23,91	755.752	100,00

* Teste de tendência do x² (< 0,0001); N: Norte; NO: Nordeste; CO: Centro-Oeste; SE: Sudeste; S:Sul.

Fonte: Dados extraídos do Sistema de Informações Hospitalares do Sistema Único de Saúde (DATASUS)^13^.

No que tange ao ano de realização do procedimento, 2014 foi responsável pelo maior número de intervenções cirúrgicas para varizes, com 79.212 (12,23%); já 2020 foi o ano em que esse tratamento foi menos empregado, totalizando 27.380 (4,22%) casos (Figura 1 e Tabela 2).

**Figura 1:** Número de cirurgias de varizes no período de 2010 a 2020, conforme ano do procedimento. Fonte: Dados extraídos do Sistema de Informações Hospitalares do Sistema Único de Saúde (DATASUS)^13^.

**Tabela 2:** Distribuição dos casos notificados no Brasil de cirurgia de varizes por região entre os anos de 2010 e 2020.

**Região**	**2010**	**2011**	**2012**	**2013**	**2014**	**2015**	**2016**	**2017**	**2018**	**2019**	**2020**	Total*	**%**
**Norte**	1.926	2.453	2.638	5.786	2.972	2.119	1.386	1.318	2.679	911	419	24.607	3,26
**Nordeste**	11.321	10.235	9.652	9.970	9.199	7.101	6.964	6.972	7.806	8.046	3.291	90.557	12,01
**Centro-Oeste**	4.056	4.856	4.523	4.457	4.270	3.857	3.404	3.309	3.140	3.304	1.487	40.663	5,39
**Sudeste**	39.510	40.389	38.751	37.516	43.333	39.282	41.290	39.304	43.016	39.582	15.912	417.885	55,43
**Sul**	18.282	17.024	17.807	17.769	19.438	16.355	15.879	16.226	18.308	16.885	6.271	180.244	23,91
**Total**	75.095	74.957	73.371	75.498	79.212	68.714	68.923	67.129	74.949	68.728	27.380	753.956	100,00

* Teste de tendência do x² (< 0,0001).

Fonte: Dados extraídos do Sistema de Informações Hospitalares do Sistema Único de Saúde (DATASUS)^13^.

Em relação à mortalidade (Tabela 3), foram registradas 40 mortes pelo procedimento no período analisado, das quais 26 ocorreram em cirurgias bilaterais (65%). No que tange às regiões, o Sudeste apresentou 24 mortes (60%), seguido pelo Nordeste, com 9 casos (22,5%). O Norte foi a região com menos óbitos no período, sendo responsável por 1 caso (2,5%).

**Tabela 3:** Distribuição dos casos notificados no Brasil de mortalidade em cirurgias de varizes por região entre os anos de 2010 e 2020.

**Cirurgia de varizes**	**N**	**%**	**NO**	**%**	**CO**	**%**	**SE**	**%**	**S**	**%**	Total*	**%**
**Unilateral**	0	0	6	42,85	0	0	7	50	1	7,14	14	35
**Bilateral**	1	3,84	3	11,53	3	11,53	17	65,38	2	7,69	26	65
**Total**	1	2,5	9	22,5	3	7,5	24	60	3	7,5	40	100,00

* Teste de tendência do x² (<0,05); N: Norte; NO: Nordeste; CO: Centro-Oeste; SE: Sudeste; S:Sul.

Fonte: Dados extraídos do Sistema de Informações Hospitalares do Sistema Único de Saúde (DATASUS)^13^.

Sobre o caráter do procedimento, as cirurgias eletivas foram maioria, representando 699.797 (92,59%) do total, sendo 266.140 (90,97%) das unilaterais e 433.657 (93,61%) das bilaterais. A região Sudeste foi responsável pela maior porcentagem de cirurgias eletivas (94,61%) em relação ao total, enquanto o Norte apresentou a menor porcentagem, com 88,25% de procedimentos realizados em caráter eletivo (Tabela 4 e Figura 2).

**Tabela 4:** Distribuição dos casos notificados no Brasil de cirurgia de varizes conforme o caráter de atendimento e região entre os anos de 2010 e 2020.

**Cirurgia para varizes**	**Caráter do atendimento**	**N**	**NO**	**CO**	**SE**	**S**	Total*	**%**
**Unilateral**	Eletiva	4.745	33.060	3.170	121.490	103.675	266.140	90,97
	Urgência	1.131	3.444	929	7.684	13.210	26.398	0,90
**Bilateral**	Eletiva	17.019	50.240	33.493	274.767	58.138	433.657	93,61
	Urgência	1.765	4.124	3.152	14.850	5.666	29.557	6,38
**Total**	Eletiva	21.764	83.300	36.663	396.257	161.813	699.797	92,59
	Urgência	2.896	7.568	4.081	22.534	18.876	55.955	7,40

* Teste de tendência do x² (< 0,0001); N: Norte; NO: Nordeste; CO: Centro-Oeste; SE: Sudeste; S:Sul.

Fonte: dados extraídos do Sistema de Informações Hospitalares do Sistema Único de Saúde (DATASUS)^13^.

**Figura 2:** Coeficiente de incidência da cirurgia de varizes por 10.000 habitantes no Brasil de 2010 a 2020. Fonte:Baseado nos dados do Sistema de Informações Hospitalares do Sistema Único de Saúde (DATASUS)^13^.

## DISCUSSÃO

A cirurgia de varizes é realizada em todas as regiões do país, sendo um procedimento disponibilizado pelo SUS nos principais hospitais regionais e naqueles com capacidade para a realização desse serviço, seguindo seus princípios e diretrizes^9^^,^^10^. Portanto, é um procedimento de suma importância, uma vez que a insuficiência venosa crônica pode levar à formação de úlceras de difícil cicatrização com possibilidade de complicações irreparáveis^12^.

As regiões com maior número de cirurgias de varizes realizadas nos últimos 10 anos foram o Sudeste, o Sul e o Nordeste. O Norte pouco contribuiu para o quantitativo dos casos, devido aos pouco mais de 24 mil procedimentos, equivalendo a 3,26% dos casos. Por outro lado, o Sudeste foi responsável por 55,41% dos procedimentos realizados. Isso demonstra fortes indícios da dificuldade de acesso e do próprio serviço especializado, principalmente em regiões mais distantes dos centros metropolitanos do país, como nos estados do Norte. Tal fato decorre possivelmente também pela maior concentração de médicos especializados nas mesmas regiões onde os serviços de média e alta complexidade estão mais disponíveis. A meta estipulada é de 2,5 médicos por 1.000 habitantes, mas, nos estados do Rio de Janeiro e São Paulo, são encontrados aproximadamente 4,44 e 3,31 médicos por 1.000 habitantes, respectivamente. Nos estados do Norte, entretanto, poucos estão próximos de alcançar o mínimo, segundo o Conselho Federal de Medicina (CFM)^14^^-^16. Quando se observa a quantidade de especialistas, essa discrepância fica mais evidente, sendo 3,1 cirurgiões vasculares para 100 mil habitantes em São Paulo e 0,5 cirurgiões vasculares por 100 mil habitantes no estado do Pará^11^^,^^15^^-^17.

Ao verificar o tipo de cirurgia, unilateral ou bilateral, constatou-se que, em todas as regiões, a cirurgia bilateral foi a mais realizada, exceto na região Sul, na qual a cirurgia unilateral representou 64,68% das operações realizadas. O achado corrobora o estudo de Defty et al.^18^, uma vez que os pacientes preferem realizar apenas uma única sessão cirúrgica (bilateral) e corrigir as veias varicosas, em vez de retornar e realizar o procedimento novamente, se submetendo a riscos anestésicos, internações e cuidados pós-operatórios.

Em relação ao período da realização do procedimento, nota-se que os anos de 2014 e 2020 apresentaram maior e menor número de execução do procedimento, respectivamente, com constante queda a partir de 2015. Assim, infere-se que, a partir do ano de 2014 a realização do procedimento cirúrgico tem diminuído, devido à implementação de tratamentos alternativos para a cirurgia de varizes no SUS, como a escleroterapia com espuma de polidocanol. Tanto por questões técnicas e administrativas quanto estruturais, acredita-se que a escleroterapia tem ganhado espaço no âmbito de correção de varizes, principalmente nos casos mais avançados, devido ao seu custo-benefício, como evidenciado em estudos recentes^19^^-^22.

A escleroterapia com espuma de polidocanol é um procedimento simples, guiado por ultrassonografia das veias superficiais e profundas dos membros inferiores, realizadas em sessões separadas para a avaliação da evolução e aplicação da espuma^20^. Esse procedimento é mais viável economicamente para os hospitais, pois a internação do paciente não é necessária, com liberação após o procedimento, custando um terço do preço da cirurgia de varizes^19^^,^^21^.

No estudo de Ramussen et al.^23^, após a comparação entre os desfechos das modalidades de tratamento, foi constatada maior taxa de recanalização e necessidade de retratamento na escleroterapia. Apesar disso, o custo médio do procedimento constatado estudo de Epstein et al. foi de apenas 249 euros, em comparação com o custo da cirurgia com ligadura alta de safena, de 894 euros^21^.

Em relação à comparação da duração da realização de cada procedimento, foi observado que a escleroterapia pode ser realizada em poucos minutos, desde a localização das veias varicosas com o ultrassom e a aplicação da espuma^20^, enquanto a flebectomia ambulatorial pode ser mais demorada, variando entre 30 minutos a 5 horas, dependendo da quantidade de veias varicosas^20^^,^^21^. Assim, a escleroterapia possibilita o tratamento de vários pacientes em um único dia em um ambulatório vascular^24^. Destaca-se a recuperação no pós-operatório, no estudo de Vernemo et al.^24^, em que foi observado que pacientes submetidos a escleroterapia tiveram ausência de dor ou mínima dor em 1 semana, com recuperação para o trabalho diário mais curta que aqueles submetidos a outros tratamentos de intervenção, possuindo a mesma qualidade de vida e recuperação após 1 ano de acompanhamento.

Em 2020, devido à pandemia causada pelo vírus SARS-CoV-2, houve um decréscimo geral na realização de procedimentos cirúrgicos não emergenciais^25^. Diversos procedimentos eletivos, além da cirurgia vascular, foram suspensos pelas autoridades de saúde durante os momentos com maior estresse sobre a rede pública no país, devido ao alto risco de exposição dos pacientes e profissionais da saúde e à necessidade de transferir recursos e alterar a infraestrutura hospitalar para tratar os pacientes infectados pelo vírus^26^. Além disso, havia receio dos pacientes em se contaminar com o patógeno, diminuindo a adesão aos procedimentos^24^^,^^27^.

Outro ponto importante a ser ressaltado é a mortalidade pelo procedimento, com 40 mortes datadas no período de 2010 a 2020, configurando uma taxa de mortalidade de 5,3 mortes por 100 mil procedimentos. A mortalidade se demostra baixa em todas as regiões, com diferenças entre seus valores relacionadas principalmente com a disparidade na quantidade da realização da cirurgia de varizes entre as regiões, com maior mortalidade no Nordeste (9,93/100 mil) e Centro-Oeste (7,37/100 mil), relacionada com a distribuição do serviço e cirurgiões vasculares pelo país^11^^,^^12^. Entre as possibilidades desencadeantes do óbito por cirurgia de varizes, estão sepse, infecção cardíaca, acidente vascular encefálico, trombose venosa profunda, tromboembolismo e embolia pulmonar, mostrando que, mesmo possuindo mortalidade baixa28, o procedimento não é isento de riscos e necessita de cuidados especializados.

O estudo de Kim et al.^29^ verificou dados semelhantes aos encontrados no presente estudo. A mortalidade encontrada foi baixa (0,02%), sem diferenças associadas com a faixa etária, apesar da propensão de idosos de sofrerem complicações e eventos adversos, diminuídos com cuidado pós-operatório. Outro estudo, em território nacional, verificou uma taxa de 4,52 procedimentos por 10 mil habitantes, com uma taxa de mortalidade muito baixa, 0,0056%^30^.

Além disso, sobre o caráter do procedimento, em todas as regiões a maior prevalência foi de cirurgias eletivas, pois são procedimentos cadastrados e ofertados pelo SUS. Isso se deve ao fato de que nem mesmo a insuficiência venosa com presença de úlceras se caracteriza como urgência, sendo classificada como passível de procedimento eletivo^31^.

A principal limitação do presente trabalho é a subnotificação presente em todos os desenhos de estudos com base em banco de dados secundários, principalmente nas regiões remotas e distantes das grandes capitais, onde há certamente uma demanda reprimida pela ausência de assistência médica especializada e que, portanto, não há como notificar, criando uma lacuna assistencial. Destaca-se, como viés de subinformação, os procedimentos realizados através da assistência privada, os quais não são notificados na base de dados utilizada nesta pesquisa. Dessa forma, os resultados obtidos podem não refletir os achados encontrados nessa população que, em geral, possui nível socioeconômico e cultural diferente da população assistida pela rede pública de saúde^1^^,^^11^.

Além disso, devem ser ressaltadas possíveis classificações errôneas, principalmente relacionadas à causa do óbito por cirurgia de varizes, pois podem ser causadas tanto por procedimentos ou ações que não estejam relacionados com a cirurgia de varizes. Outra limitação presente durante a realização do estudo foi a ausência de dados acerca da escleroterapia não estética no DATASUS, limitando possíveis cruzamentos de dados e correlações com a flebectomia ambulatorial.

O estudo visou traçar o perfil da cirurgia para o tratamento de varizes, verificando suas características e região de realização, demonstrando a relação entre diversos fatores com a realização da cirurgia de varizes e verificando possíveis motivos para a redução da sua realização ao longo do tempo, bem como características de sua técnica.

## CONCLUSÃO

Neste estudo, foi possível identificar que as cirurgias de varizes são em sua grande maioria bilaterais, eletivas e concentradas nas regiões Sul e Sudeste. Infere-se que a implementação de tratamentos alternativos mais acessíveis, como a escleroterapia, justifique a diminuição gradual da realização dos procedimentos cirúrgicos ao longo dos anos.
